# A Robust Fabric Defect Detection Method Based on Improved RefineDet

**DOI:** 10.3390/s20154260

**Published:** 2020-07-30

**Authors:** Huosheng Xie, Zesen Wu

**Affiliations:** School of Mathematics and Computer Science, Fuzhou University, Fuzhou 350108, China; N180320067@fzu.edu.cn

**Keywords:** fabric defect detection, object detection, improved RefineDet, Full Convolutional Channel Attention block, Bottom-up path augmentation Transfer Connection Block, DIoU-NMS, cosine annealing scheduler

## Abstract

This paper proposes a robust fabric defect detection method, based on the improved RefineDet. This is done using the strong object localization ability and good generalization of the object detection model. Firstly, the method uses RefineDet as the base model, inheriting the advantages of the two-stage and one-stage detectors and can efficiently and quickly detect defect objects. Secondly, we design an improved head structure based on the Full Convolutional Channel Attention (FCCA) block and the Bottom-up Path Augmentation Transfer Connection Block (BA-TCB), which can improve the defect localization accuracy of the method. Finally, the proposed method applies many general optimization methods, such as attention mechanism, DIoU-NMS, and cosine annealing scheduler, and verifies the effectiveness of these optimization methods in the fabric defect localization task. Experimental results show that the proposed method is suitable for the defect detection of fabric images with unpattern background, regular patterns, and irregular patterns.

## 1. Introduction

In the textile industry, defect detection is an important part of the quality control of fabric products. It aims to detect fabric products with defective areas efficiently and timely to reduce additional economic losses caused by these low-quality products. Many textile mills still use the manual inspection method to detect defects [1], which causes inspectors to inadvertently miss some products with defects after a prolonged observation. To this end, it is necessary to develop an automatic defect detection method based on computer vision.

Since the 1980s, there have been a lot of works in the field of automated defect detection. Among them, the traditional works are mainly divided into the following categories: (1) Spectral-based methods, (2) statistical-based methods, (3) structural-based methods, and (4) traditional learning-based methods. For example, Jia et al. [2] proposed a fabric defect detection method based on lattice segmentation and the Gabor filtering. The method mainly used the Gabor filter band, a kind of spectral-based method, to extract features of the image patch (i.e., semantic lattice). Jing et al. [3] proposed two defect detection methods, i.e., Gabor preprocessed Golden Image Subtraction (GGIS), and Gabor preprocessed Golden Image Subtraction Based on a Genetic Algorithm (GAGIS), and the two methods mainly used the Gabor filter to preprocess the input image and used the image subtraction technique to locate the defective area. Pan et al. [4] and Zhang et al. [5] used the mathematical morphology method, a kind of Statistical-based method, to enhance the defective area in unpatterned images. Liu et al. [6] proposed a defect detection method proposed based on unsupervised segmentation (a dictionary learning method based on image patch) and Extreme Learning Machine (ELM), and the method mainly extracted geometric features (using Hu invariant moment method) and texture features (using an optimal wavelet packet technology) for ELM classifier. These traditional defect detection methods can get good results on some specific fabric products (unpatterned or regular patterned background). However, most of them are mainly based on predefined features or hand-craft features [7], including statistical features, structural features, and spectral features of the images. This means that the configuration of model parameters requires some prior knowledge or problem-specific research. When the fabric products with new design patterns appear, these detection methods must be modified or even redesigned [8].

With the development of deep Convolution Neural Networks (CNN) and Graphics Processing Units (GPU) computing power, many researchers pay more attention to studying an efficient defect detection method based on deep learning. Different from traditional methods, these deep learning methods mainly use the CNN, one of the most typical representative of deep neural networks, for feature extraction. Through a series of convolution, activation, and pooling operations, the CNN network can adaptively generate a hierarchy of features of the input image (i.e., feature maps) [9]. With this powerful feature learning capability, deep learning models based on CNN have been widely applied to various sub-fields of defect detection, such as fabric defect detection, lithium battery defect detection. For example, Zhao et al. [7] proposed a fabric defect classification method using a three-parallel-module integrated structure (i.e., Stacked Convolutional Auto-Encoders (SCAE), a shallow CNN and a deep CNN combining non-local block). Gan et al. [9] designed a joint detection CNN architecture, which contains two major parts: The global detection part (used for background classification in image level) and the sub detection part (used for defect classification in image patch level). Mei et al. [10] proposed an unsupervised learning approach based on a convolutional denoising autoencoder at multiple Gaussian pyramid levels (Multi-scale CDAE). Chen et al. [11] proposed a weakly-supervised learning-based surface defect classification and segmentation framework based on CNN with the Spatial Attention Mechanism (SAM). Hu et al. [12] proposed an unsupervised fabric defect detection method based on Deep Convolutional Generative Adversarial Network (DCGAN).

As a branch of deep learning, more and more object detection models based on CNN have been used in recent defect detection research, including the two-stage detector (Faster RCNN [13]) and the one-stage detector (SSD [14], YOLOv2 [15], YOLOv3 [16]). For example, Liu et al. [17] and Liu et al. [18] used Faster RCNN as the base model. [17] applied some data augmentation methods and soft NMS to further improve the performance of the model, and [18] combined the high-level features of the ROI pooling layer output with the low-level features obtained by the Histogram of Oriented Gradient (HOG) in the original Faster RCNN. Wu et al. [19] designed a Composite Interpolating Feature Pyramid Network (CI-FPN) as the neck structure and introduced a guided anchor mechanism and position-sensitive RoI-Align in head structure. Liu et al. [20] introduced SSD to the defect detection for the first time and added the third-level feature conv3_3 to the feature pyramid to achieve the detection of small objects. Zhang et al. [21] verified the performance of three variants of YOLOv2 models and proposed a yarn-dyed fabric defect automatic localization and classification method. Wei et al. [22] designed three models based on YOLOv3 to detect the surface defects on magnetic tiles. Jing et al. [8] introduced the YOLOv3 model to fabric defect detection and added a lower feature layer to the feature pyramid.

The advantages of the fabric defect detection methods based on object detection model are summarized as follows:

- **Powerful feature extraction capability.** Object detection uses deep convolutional neural networks as the backbone [23], which can automatically extract the defect features of the input image.
- **Efficient neck structure (feature pyramid and feature fusion) structure.** Most object detection models have a neck (i.e., feature pyramid and feature fusion), which can detect defect areas with different sizes in the image. These feature pyramid structures are roughly divided into the following categories [24,25]: (1) SSD-style, (2) FPN-style, (3) STDN-style, (4) M2Det-style, (5) PAN-style. Some recent studies have made improvements on the neck structure and achieved a good detection result [19,26].
- **Flexible selection of the model.** Pre-existing object detection models usually can be divided into two categories, the one-stage object detection models and the two-stage object detection models. In general, the two-stage object detection models have higher localization and object classification accuracy and the one-stage object detection models are time-efficient and can be used for real-time detection [27]. According to the needs of different application scenarios, we can choose an appropriate model for training. In the field of fabric defect detection, the one-stage object detector can be selected as the base model to meet the needs of real-time detection.
- **Classification and localization results based on image patch level.** The object detection model generates candidate object bounding boxes (i.e., image patches with defect area or background area) from the feature maps and sends them to the classification subnetwork and regression subnetwork, respectively. After Non-Maximum Suppression (NMS), we can directly get the defect categories and location of each predicted defect image patch.
- **Various general optimization methods.** In the whole training and testing stages of the object detection model, many researchers proposed various general optimization methods [25,27,28], including data augmentation method, attention mechanism, learning rate scheduling strategy, activation function selection, loss function optimization, and post-processing method improvements. These optimization methods can boost the performance of all popular object detection models without introducing extra computational cost during inference [28].
- **Good generalization ability.** The object detection model mainly learns the feature of defect objects, rather than the background. Therefore, when there are enough training images with defect objects, it can be suitable for fabric defect detection under different texture backgrounds. Meanwhile, some state-of-the-art data augment [25,29,30,31], and Weakly Supervised Object Localization (WSOL) [32,33] methods can alleviate the problems of insufficient training samples and expensive manual labeling to a certain extent.

In view of this, a fabric defect detection method based on an improved RefineDet is proposed in this paper. Firstly, we use a one-stage object detection model, RefineDet [34], as the base model, which has the advantages of both one-stage and two-stage object detection models. Secondly, we design an improved head structure combing Fully Convolutional Channel Attention (FCCA) block and Bottom-up path Augmentation Transfer Connection Block (BA-TCB), and this improved head structure can effectively improve the model’s ability to locate defective areas. Finally, we apply and verify the influence of many general optimization methods in the field of fabric defect detection, which can boost the performance of the proposed defect detection model without introducing too much extra computational cost during inference. On the three public defect datasets TILDA, Hong Kong Patterned Textures Database, and DAGM2007, the mAP and F1-score metrics of the proposed method exceed 80%. Experimental results show that the proposed method is suitable for the defect detection of fabric images with unpattern background, regular patterns, and irregular patterns and can detect different fabric defects at high speed.

The main contributions of this work are summarized as follows:

- We use RefineDet as the base model of defect detection. To the best of our knowledge, this is the first time that RefineDet has been used for fabric defect detection. Using the special two-step classification and regression structure of RefineDet, the proposed method can better detect the defect area compared with other common object detectors.
- We design an improved head structure. This improved head structure consists of Fully Convolutional Channel Attention-based Anchor Refinement Module (FCCA-ARM), BA-TCB, and Object Detection Module (ODM). By adding the channel attention mechanism (i.e., FCCA block) and designing the bottom-up path augmentation structure (i.e., BA-TCB), the detection accuracy of the proposed method is further improved.
- We research and verify the influence of many general optimization methods in the field of fabric defect detection. The state-of-the-art general optimization methods, such as attention mechanism, DIoU-NMS, and cosine annealing scheduler, are successfully applied to our detection model, which is an important reference for researchers in the fabric defect detection field.

The remainder of this paper is organized as follows: Related work is introduced in Section 2. The details of our proposed method are included in Section 3. Experiment results and discussion are provided in Section 4. Finally, Section 5 is the conclusion of this work.

## 2. Related Work

This section first shows the structure of the object detection model, then introduces some state-of-the-art general optimization methods, and finally summarizes the advantages of RefineDet, the base object detector used in our work.

### 2.1. The Structure of The Object Detection Model

In recent years, the object detection model has formed a fixed structural framework. An object detection model is composed of several parts:

- **Acquisition of input images.** The raw images used for recent research were mainly from public datasets or collected by textile factories and laboratories. Some typical public defect detection datasets are TILDA dataset (https://lmb.informatik.uni-freiburg.de/resources/datasets/tilda.en.html), DAGM2007 dataset (https://hci.iwr.uni-heidelberg.de/content/weakly-supervised-learning-industrial-optical-inspection), and Hong Kong patterned texture database (https://ytngan.wordpress.com/codes/); and some self-built datasets are DHU-FD-500 [7], DHU-FD-1000 [7], lattice [8], FDBF dataset [19], etc.
- **Image preprocessing.** Insufficient of defect samples is a challenge in the research of fabric defect detection methods. To address this problem, the techniques of image pyramid [35] or sliding window [9] are introduced in the stage of image preprocessing. Particularly, many data augmentation methods based on photometric distortion and geometric distortion are widely used to increase the variability of the input images, including adjusting the brightness, contrast, hue, saturation, and noise of input images, image scaling, cropping, flipping, and rotating.
- **Backbone.** The backbone as the basic feature extractor of the object detection task is used to generate the output feature maps of the corresponding input images. The common backbones are VGG-16 [34,36], ResNet [37,38], ResNeXt [39], DarkNet-19 [15,21], DarkNet-53 [8,16], MobileNet [40,41], and ShuffleNet [42].
- **Neck structure.** Object detection model developed in recent years often insert the neck structure between the backbone network and the head structure, and the neck structure is usually used to collect feature maps from different layers with different resolutions of the backbone [25]. Common neck structures in recent research are Feature Pyramid Network (FPN) [43] with its variants CI-FPN [19], BI-FPN [26], NAS-FPN [44], and Path Aggregation Network (PAN) [45].
- **Head.** The head is used to predict classes and bounding boxes (detection results in image patch level) of defect objects. In this stage, one-stage object detectors directly predict class and bounding boxes from dense candidate boxes. Two-stage object detection models first filter out sparse refined boxes from dense boxes and then predict the results from the refined boxes. Therefore, the two-stage models have higher accuracy, and the one-stage models are time-efficient.
- **Post-processing.** In the testing stage, the post-processing step deletes any weak detecting results [23]. For example, NMS is a widely used method that only remains the object boxes with the highest classification score in predicted results. The common NMS methods are greedy-NMS [46], soft-NMS [47], adaptive-NMS [48].

### 2.2. The State-of-The-Art General Optimization Method

Recently, many works have proposed new components and optimization methods in the object detection model, and have achieved remarkable results in the general domain. These optimization methods cover all training and testing stages of the detector, including image preprocessing stage, network construction (attention mechanism, activation function), loss function design, and post-processing stage.

In the image preprocessing stage, many novel data augment methods have appeared, such as Mosaic [25], MixUp [29], CutOut [30], CutMix [31], and Generative Adversarial Network (GAN) [49]. In the network construction stage, many attention mechanisms can be combined with the network’s backbone, neck, and head to get better localization results, such as channel-wise attention (Squeeze-and-Excitation (SE) block [50], Channel Attention (CA) block [51]) and point-wise attention (SAM [51]). For example, on the MS COCO dataset, Woo et al. [51] have proved through experiments that the Faster RCNN (the backbone is ResNet-50) combining the CA block and SAM can improve mAP metric by 2% compared with the original Faster RCNN. On the VOC2007 testing set, when combining CA block and SAM, the StairNet framework (the backbone is VGG-16), one of the strongest multi-scale methods based on SSD, can improve the mAP metric by 1.5% compared with the original SSD. When combining the SE block, the StairNet framework can improve the mAP metric by 1.3% compared with the original SSD. Meanwhile, some new activation functions are designed to optimize the CNN model, such as Swish [52], Mish [53]. In addition, Zheng et al. [54] designed an improved IoU loss function as the regression loss function and used DIoU-NMS for the object detection model in the post-processing stage.

Inspired by these optimization methods, we introduce some state-of-the-art general optimization methods to this work and verify the influence of these optimization methods on the fabric defect detection performance.

### 2.3. The Overview of RefineDet

In the head structure, the two-stage object detection models have a densely tailing process to obtain as many as candidate boxes, and this process is often time-consuming and inefficient. It is a hot trend to develop an efficient object detection method which can eliminate so much redundancy while maintaining high accuracy [23]. To address this issue, Zhang et al. [34] design a new one-stage framework (we call it RefineDet) to inherit the advantages of the one-stage detector and two-stage detector.

**Figure 1:**
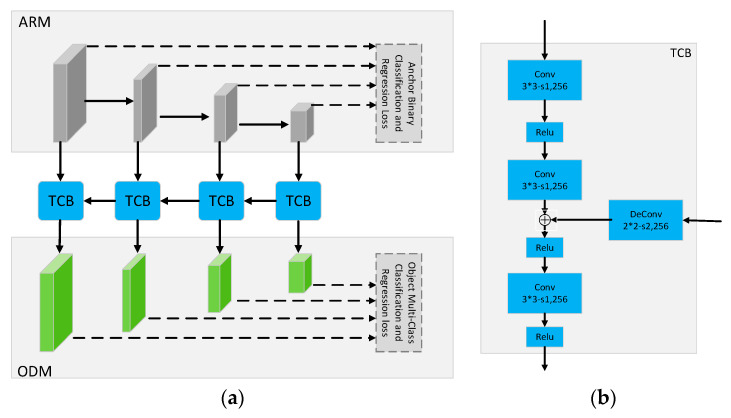
The main structure of the original RefineDet. (**a**) The ARM, TCB, and ODM of original RefineDet; (**b**) Description of TCB structure.

RefineDet uses VGG-16 or ResNet-101 as the backbone for feature extraction and integrates the neck structure (feature pyramid and feature fusion) into the head structure. The head structure of RefineDet consists of Anchor Refinement Module (ARM), Transfer Connection (TCB), and ODM (As shown in Figure 1):

- **ARM.** The four feature maps of ARM mainly come from different layers in the backbone. The ARM is designed to coarsely filter out refined boxes from dense candidate boxes and adjust the localizations and sizes of refined boxes (i.e., the first step classification and regression) so as to provide better initialization for the subsequent multi-class classification and regression task.
- **TCB.** TCB aims to transfer the refined boxes to ODM and integrate different features information (feature fusion) of shallow layers and deep layers of ARM.
- **ODM.** ODM takes the refined boxes as the input from the TCB and outputs predicted multi-class labels and the localizations of refined boxes (i.e., the second step classification and regression). In the testing stage, we can get the predicted results in the image patch level after NMS processing.

As a kind of one-stage object detection model, the RefineDet also has the characteristics of two-stage models (i.e., Two-step cascaded regression and two-step classification), which can better predict hard detected objects, especially for small objects and get more accurate locations of objects [23]. RefineDet achieves 85.8% and 86.8% mAP on VOC2007 and VOC2012, two public general datasets, with the VGG-16 backbone. Meanwhile, it runs at 40.2 FPS with the input sizes 320 × 320. Considering these advantages of RefineDet, we use it as the base model in our fabric defect detection method.

## 3. Methodology

**Figure 2:**
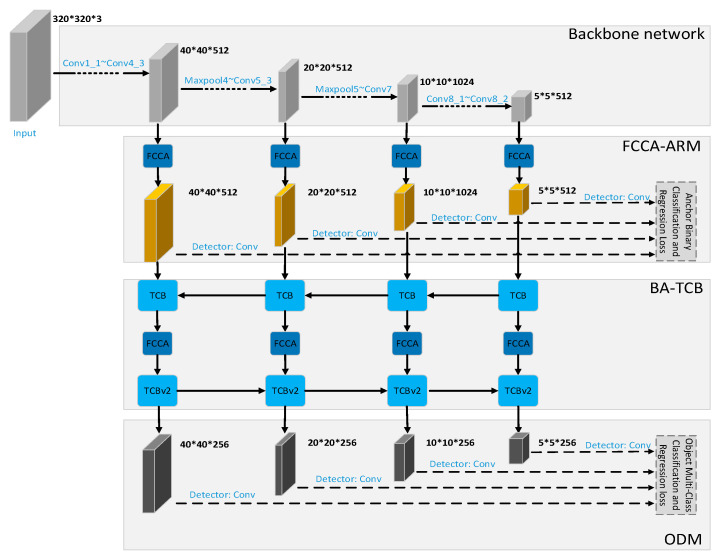
The network architecture of the proposed method.

As shown in Figure 2, we propose a robust fabric defect detection method based on improved RefineDet. The improved RefineDet network is consist of the following part: VGG-16 based backbone, FCCA-ARM, BA-TCB, ODM. In addition, many general optimization methods, such as mosaic data augmentation, attention mechanism, DIoU-NMS, and cosine annealing scheduler are applied to the training and testing stage of our model, which effectively improves the defect localization accuracy of the proposed method.

### 3.1. Data Augmentation

In general, neural networks need to train millions of parameters. The premise of making these parameters work correctly is enough input data for training. However, in fabric defect detection, the data is not as much as we think. The size of the fabric datasets is dozens to thousands. Insufficient of defect sample is a challenge in the research of fabric defect detection methods.

To address the issue above, we mainly use data augmentation methods in the image preprocessing stage. In addition to using some data augmentation methods based on photometric distortion and geometric distortion, we also use the mosaic method in our work.

**Figure 3:**
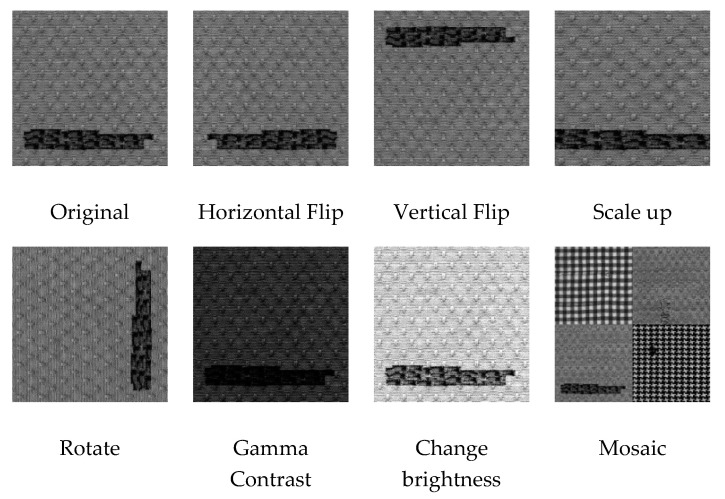
Data augmentation methods.

As shown in Figure 3, we can perform a combination of various data augmentation methods (i.e., image flip, rotation, scale-up, brightness change, contrast change, and mosaic) to increase the number of training images. In addition, when the generated images are close to the natural image, these data augmentation methods can increase the diversity of the input images, which makes the trained model more robust.

### 3.2. VGG-16 Based Backbone

We do not change the backbone of the original RefineDet, i.e., we use VGG-16 as the base feature extract network, remove fc8, replace fc6, fc7 into convolution layers (Conv6, Conv7) and add two extra convolution layers (Conv8_1, Conv8_2) after VGG-16. The configuration of each layer of the backbone network can be seen in Table 1. Between Conv1_1 and Conv7, all convolution layers are followed by the Relu activation function.

### 3.3. The Improved Head Structure

Inspired by BI-FPN [26], PANet [45], and CBAM [51], we design an improved head structure to improve the defect localization ability of the proposed method. The head in our model is consists of three parts: Fully convolutional Channel Attention-based Anchor Refinement Module (FCCA-ARM), Bottom-up path Augmentation Transfer Connection Block (BA-TCB), and ODM.

#### 3.3.1. FCCA-ARM

The Layers in the feature pyramid come from Conv4_3, Conv5_3, Conv7, and Conv8_2 of the backbone network. By using the attention mechanism, the model can focus on important features and suppress unnecessary ones [51]. Therefore, we design an improved ARM that uses fully convolutional channel attention blocks to process those layers before ARM.

**Figure 4:**
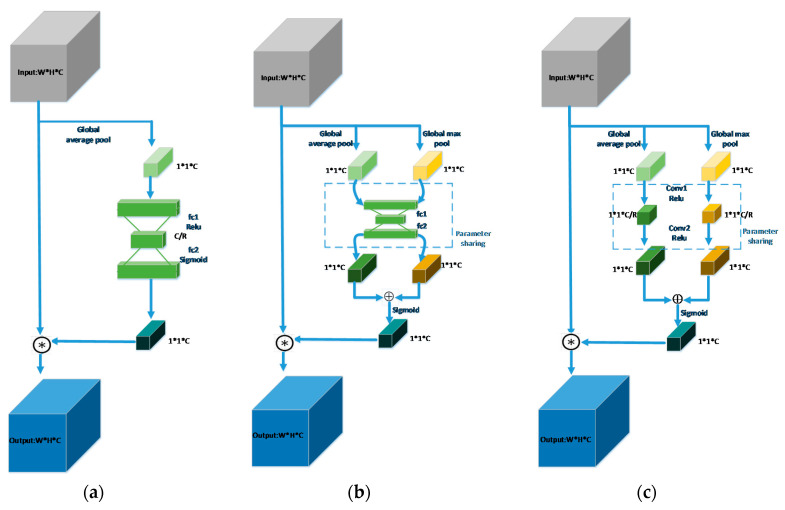
Different channel attention blocks. (**a**) SE block; (**b**) CA block; (**c**) the Full Convolutional Channel Attention (FCCA) block.

As seen in Figure 4c, given an intermediate feature map $X\in {\mathbb{R}}^{W*H*C}$ as input, the output Y of fully convolutional channel attention block is computed as Equation (1).

(1) $$Y=\sigma_{1}\left(FConv\left(AvgPool\left(X\right)\right)+FConv\left(MaxPool\left(X\right)\right)\right)$$

in which,

(2) $$FConv\left(*\right)=\sigma_{2}\left(Conv2\left(\sigma_{2}\left(Conv1\left(*\right)\right)\right)\right)$$

AvgPool(∗) is the global average pool operation. MaxPool(∗) is the global max pool operation. Conv1(∗) is convolution layer with 1∗1-s1-p0-C/R (i.e., kernel size is 1∗1, the stride is 1, padding is 0, output channel is C/R, R is the reduction ratio). Conv2(∗) is the convolution layer with 1∗1-s1-p0-C. $\sigma_{1}\left(*\right)$ is the Sigmoid activation function. $\Sigma_{2}\left(*\right)$ is the Relu activation function.

Different from the SE block (see Figure 4a), the channel attention mechanism uses both average-pooled and max-pooled features simultaneously. In addition, we use two convolutional layers instead of the multilayer perceptron (MLP) in the original channel attention block (see Figure 4b), which can increase representation power and decrease the parameters of our model.

#### 3.3.2. BA-TCB

Feature maps in deep layers often contain more global context information, while other feature maps in shallower layers contain more local textures and pattern structures. Thus, FPN adds a top-down path to propagate these semantically strong features. PANet adds an additional bottom-up pathway on top of the FPN. This bidirectional (top-down and bottom-up) structure can be used widely in BI-FPN, CI-FPN and have proved effective in practical applications.

Inspired by this bidirectional structure, we design four new blocks, TCBv2, to form a bottom-up pathway and add four fully convolutional channel attention blocks between TCB and TCBv2.

**Figure 5:**
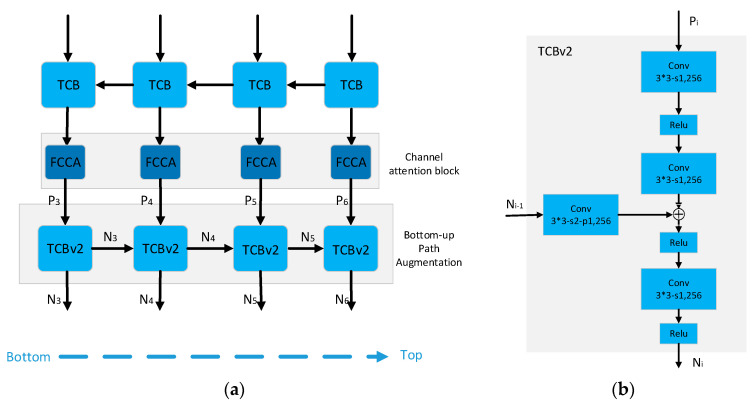
The structure of BA-TCB. (**a**) Bidirectional structure (Top-down TCB and Bottom-up TCBv2); (**b**) The structure of TCBv2.

As seen in Figure 5, the four input of TCBv2 is denoted as $\left(P_{3},P_{4},P_{5},P_{6}\right)$, and the output feature maps $\left(N_{3},N_{4},N_{5},N_{6}\right)$ are computed as Equations (3) and (4).

(3) $$N_{3}=TCBv2\left(\mathrm{zeros},P_{3}\right)$$

(4) $$N_{i}=TCBv2\left(N_{i-1},P_{I}\right),\;i=4,5,6$$

where I refers to a feature level with a resolution of 12i of the input images (e.g., $P_{3},N_{3}$ are feature maps with resolution 40∗40 when the input image is 320∗320).

#### 3.3.3. ODM

ODM is consists of four convolutional layers with channel k∗(C+4). Where C is the number of defect classes for the multi-classification task, 4 refers to the four coordinates value (i.e., x_min_, y_min_, w, h) of predicted boxes for the regression task. K refers to the number of aspect ratios, and in our model k is set to 3, which means that the aspect ratios are 1:1, 1:2, and 2:1.

### 3.4. The Two-Step Loss Function and DioU-NMS

Similar to original RefineDet, in the training stage, the total two-step loss function is designed as Equation (5).

(5) $$L\left(\left\{p_{i},X_{i}\right\},\left\{c_{i},B_{i}\right\}\right)=arm\_c\left(\left\{p_{i}\right\}\right)+arm\_l\left(\left\{x_{i}\right\}\right)+odm\_c\left(\left\{c_{i}\right\}\right)+odm\_l\left(\left\{t_{i}\right\}\right)$$

in which,

(6) $$arm\_c\left(\left\{p_{i}\right\}\right)=\frac{1}{N_{arm}}\left(\underset{i}{\sum }\left[l_{i}^{gt}\geq 1\right]\times CE\left(p_{i},l_{i}^{gt}\right)\right)$$

(7) $$arm\_l\left(\left\{X_{i}\right\}\right)=\frac{1}{N_{arm}}\left(\underset{i}{\sum }\left[l_{i}^{gt}\geq 1\right]\times L1\left(X_{i},B_{i}^{gt}\right)\right)$$

(8) $$odm\_c\left(\left\{c_{i}\right\}\right)=\frac{1}{N_{odm}}\left(\underset{i}{\sum }\left[l_{i}^{gt}\geq 1\right]\times CE\left(c_{i},l_{i}^{gt}\right)\right)$$

(9) $$odm\_l\left(\left\{B_{i}\right\}\right)=\frac{1}{N_{odm}}\left(\underset{i}{\sum }\left[l_{i}^{gt}\geq 1\right]\times L1\left(B_{i},B_{i}^{gt}\right)\right)$$

I is the index of anchor boxes in a training batch. $p_{i},\;X_{i}$ are the predicted defect class confidence being a defect object and localization coordinates of the anchor box i in the CA-ARM structure, respectively. $c_{i},\;B_{i}$ are the predicted object class confidence and coordinates of the refined box in the ODM, respectively. $N_{arm}$, $N_{odm}$ are the numbers of defect class boxes in the CA-ARM and ODM, respectively. $l_{i}^{gt}$ is the ground truth class label of anchor box i. $B_{i}^{gt}$ is the ground truth localization coordinates. The indicator function $\left[l_{i}^{gt}\geq 1\right]$ means that when the box i belongs to the defect class, it outputs 1, otherwise 0. In arm_c, CE is the cross-entropy loss over two classes (defect and background), and in odm_c, CE(∗) is the cross-entropy function over all defect classes. L1(∗) is the Smooth L1 function.

After calculating the total loss, backpropagation is performed to update the model parameters. After the specified number of iterations (in our experiment, we set it 120 k), the training of model finish.

Generally, the IoU-based method can be defined as $\mathrm{IoU}-ℛ\left(B,B^{\mathrm{gt}}\right)$. The penalty term $ℛ\left(B,B^{\mathrm{gt}}\right)$ is designed for minimizing the normalized distance between predicted boxes B and the ground truth boxes $B^{\mathrm{gt}}$ and making the regression (i.e., defect area localization) more accurate and faster. The three important geometric factors that affect the accuracy of the regression of the anchor boxes are the overlapping area between B and $B^{\mathrm{gt}}$, the distance between the center points of B and $B^{\mathrm{gt}}$ and the aspect ratio of the box. Considering these factors, many improved IoU calculation methods have appeared in recent years. For example, gIoU designs a novel penalty item based on IoU to solve the problem of gradient disappearance when the bounding boxes do not overlap (as seen in Equation (10)). dIoU takes into account the factor of the distance between the center points (as seen in Equation (11)). By adding a penalty term to the original IoU, the normalized distance between the center points of the two bounding boxes is directly minimized, so that it can converge faster than the gIoU loss. Considering all three geometric factors, cIoU adds a penalty item to the aspect ratio based on the dIoU, which making regression more accurate and faster (as seen in Equation (12)).

(10) $$\mathrm{gIoU}\left(B,B^{\mathrm{gt}}\right)=\mathrm{IoU}\left(B,B^{\mathrm{gt}}\right)-{ℛ}_{\mathrm{gIoU}}\left(B,B^{\mathrm{gt}}\right)=\mathrm{IoU}\left(B,B^{\mathrm{gt}}\right)-\frac{\left|A_{c}-A_{B^{\mathrm{gt}}⋂B}\right|}{\left|A_{c}\right|}$$

(11) $$\mathrm{dIoU}\left(B,B^{\mathrm{gt}}\right)=\mathrm{IoU}\left(B,B^{\mathrm{gt}}\right)-{ℛ}_{\mathrm{dIoU}}\left(B,B^{\mathrm{gt}}\right)=\mathrm{IoU}\left(B,B^{\mathrm{gt}}\right)-\frac{\rho^{2}\left(b,b^{gt}\right)}{l_{c}{}^{2}}$$

(12) $$\mathrm{cIoU}\left(B,B^{\mathrm{gt}}\right)=\mathrm{IoU}\left(B,B^{\mathrm{gt}}\right)-{ℛ}_{\mathrm{cIoU}}\left(B,B^{\mathrm{gt}}\right)=\mathrm{IoU}\left(B,B^{\mathrm{gt}}\right)-\frac{\rho^{2}\left(b,b^{gt}\right)}{l_{c}{}^{2}}-\alpha \upsilon$$

in which,

(13) $$v=\frac{4}{\pi^{2}}{\left(arctan\frac{w^{gt}}{h^{gt}}-arctan\frac{w}{h}\right)}^{2}$$

(14) $$\alpha =\frac{v}{\left(1-\mathrm{IoU}\left(B,B^{\mathrm{gt}}\right)\right)+v}$$

$A_{c}$ is the area of the smallest closure box of $B^{\mathrm{gt}}$ and B. $A_{B^{\mathrm{gt}}⋂B}$ is the intersection area of $B^{\mathrm{gt}}$ and B. ρ(∗) is the Euclidean distance. $b,\;b^{gt}$ are the central points of $B^{\mathrm{gt}}$ and B, respectively. $l_{c}$ is the diagonal length of the smallest closure box of $B^{\mathrm{gt}}$ and B. And $\frac{w^{gt}}{h^{gt}}$, $\frac{w}{h}$ are the aspect ratio of $B^{\mathrm{gt}}$ and B, respectively.

Although cIoU is better than dIoU under normal circumstances, dIoU is better than cIoU in small object detection, i.e., the consistency of the aspect ratio may not affect the regression accuracy of small objects [54]. Therefore, we use dIoU-NMS to post-process the detection results of the proposed methods in the testing stage. For the predicted box M with the highest score, the dIoU-NMS can be formally defined as Equation (15).

(15) $$s_{i}=\{\begin{array}{cc} s_{i}, & DIoU\left(M,B_{i}\right)<T_{s}\\ 0, & DIoU\left(M,B_{i}\right)\geq T_{s} \end{array}$$

This means that these predicted defect boxes $B_{i}$ that overlap with *M* greater than the NMS threshold $T_{s}$ will be filtered.

### 3.5. Leaning Rate Adjustment Method Based on Cosine Annealing Scheduler

The common learning rate adjustment strategies mainly include the following: (1) StepLR (i.e., the learning rate in the training stage decreases according to regular intervals); (2) MultiStepLR (i.e., the learning rate decreases according to preset intervals); (3) ExponentialLR (i.e., the learning rate decreases according to exponential decay); (4) ReduceLROnPlateau (i.e., the learning rate in the training stage decreases when an indicator no longer changes); (5) CosineAnnealingLR (i.e., the learning rate in the training stage change according to the period of the cosine function). In the training stage, we use the cosine annealing scheduler to adjust the learning rate. The cosine annealing scheduler adjusts the learning rate in an intermediate iteration or epoch $\eta_{t}$ as Equation (16).

(16) $$\eta_{t}=\eta_{min}+\frac{1}{2}\left(\eta_{max}-\eta_{min}\right)\left(1+cos\left(\frac{T_{cur}}{T_{max}}\pi \right)\right)$$

where $\eta_{min}$ is the valley value of cosine function, and $\eta_{min}$ is set to 0 in our experiment. $\eta_{max}$ is the peak value of cosine function (i.e., initial learning rate), $\eta_{max}$ is set to $10^{-3}$. $T_{max}$ is half of the Cosine function period, and $T_{max}$ is set to 120 k. $T_{cur}$ refers to the number of epochs or iterations recorded since the last start.

**Figure 6:**
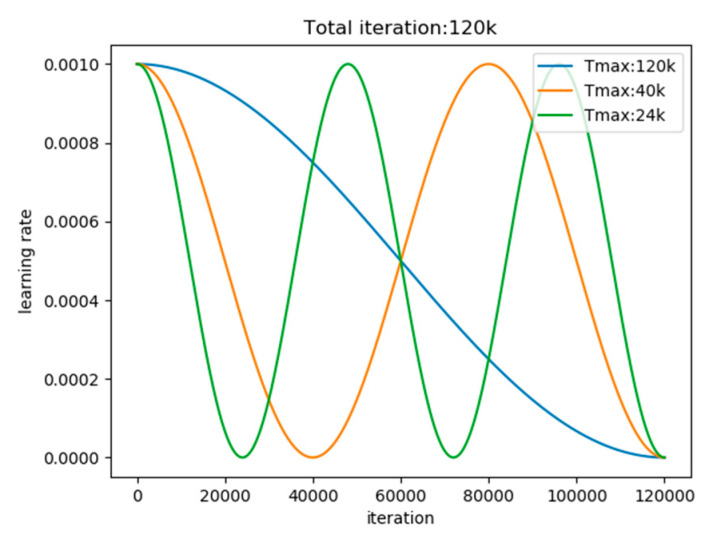
The cosine annealing leaning rate in different Tmax.

As seen in Figure 6, the cosine annealing scheduler takes the cosine function as a period and resets the learning rate at the maximum value of each period. Taking the initial learning rate as the maximum learning rate and taking 2∗ $T_{max}$ as the period, it decreases first and then rises within a period.

## 4. Results and Discussion

In this section, we will show the robust fabric defect detection ability of the proposed method through a large number of comparison experiments and ablation experiments. All experiments were performed on an LZ-748GT workstation configured with Intel E5-2600 CPU (2200 MHz), 32GB RAM, and a Nvidia 16GB TITAN XP GPU. The code and detection results of the proposed method are available at https://github.com/2089527378/Fabric-defect-detection-based-on-improved-RefineDet.

### 4.1. The Datasets and Evaluation Metrics

In order to show the robust ability of the proposed method in the fabric defect detection task, we use three representative public defect datasets (i.e., TILDA dataset, Hong Kong Patterned Textures Database, and DAGM2007 dataset) in our experiment. In addition, to better compare the improvements, we also show the performance of our method on the PASCAL VOC dataset, an object detection standard dataset and the details can be seen in Appendix A.

**TILDA dataset**

**Figure 7:**
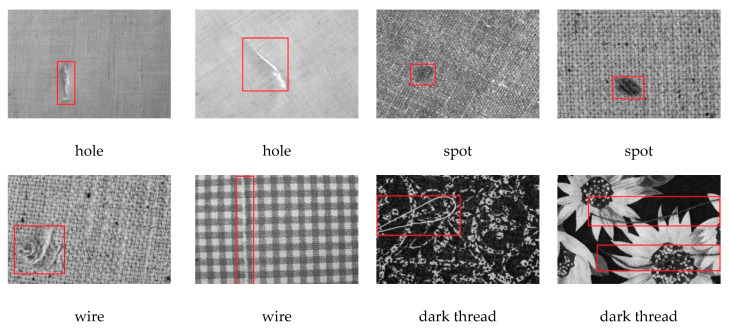
Illustration of defect images on TILDA dataset.

TILDA is a common public textile texture dataset that was developed within the framework of the working group Texture Analysis of the DFG’s (Deutsche Forschungsgemeinschaft) major research programme “Automatic Visual Inspection of Technical Objects” [35]. A total of eight representative textile kinds (covering four unpatterned fabrics, two regular patterned fabrics, and two complex irregular patterned fabrics) were included in the dataset. Based on the analysis of textile atlases, seven defect classes are defined. In order to evaluate the performance of the proposed method, we use four common defect classes of fabric defects, which are hole (E1), spot (E2), wire (E3), and dark thread (E4). An illustration of defects and texture background can be found in Figure 7. The size of the images is 768 × 512 pixels, and the number of defective images is 1597.

**Hong Kong Patterned Textures Database**

**Figure 8:**
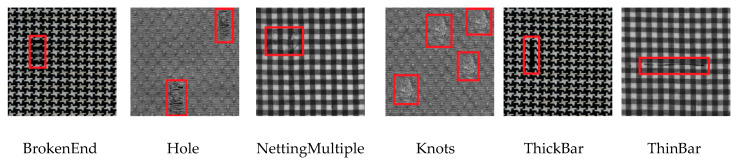
Illustration of images on Hong Kong dataset.

Hong Kong Patterned Textures Database (we call it “Hong Kong dataset”) is a regular patterned database provided by Dr. Ngan, the Research Assistant Professor, Department of Mathematics, Hong Kong Baptist University. It consists of non-defective and defective images in three regular patterned fabrics (i.e., star-patterned, box-patterned, and dot-patterned fabrics). As shown in Figure 8, Six defect classes are defined, which are BrokenEnd (bn), Hole (he), NettingMultiple (nm), Knots (kn), ThickBar (tk), and ThinBar (tb). This dataset is highly challenging because of the small number of samples (only 82 defective images in all six defect classes). The size of images on the Hong Kong dataset is 450 × 450 pixels.

**DAGM2007 Dataset**

**Figure 9:**
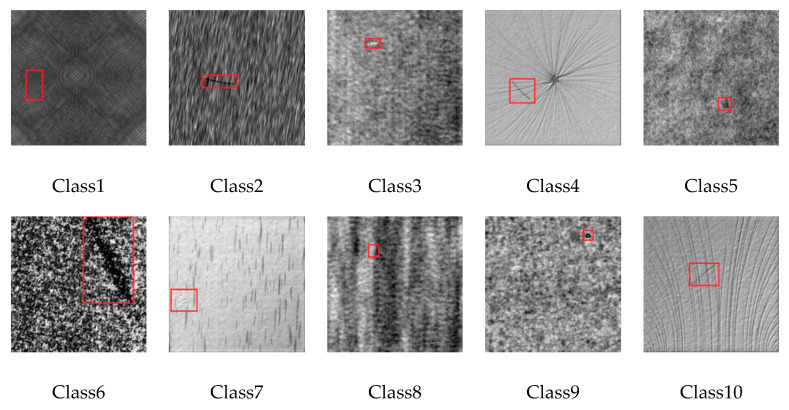
Illustration of images on DAGM2007 Dataset.

DAGM2007 is a benchmark defect dataset that is provided by the International Association for Pattern Recognition (IAPR) and the German Chapter of the European Neural Network Society (GNSS). The defect area is artificially generated, but similar to real-world problems. The texture background is mainly unpatterned or simple irregular patterned, and there are ten defect classes in DAGM2007 dataset which are Class1, Class2… Class10 (see Figure 9). The size of images is 512 × 512 pixels. The number of training defect images is 1046, and the number of testing images is 1054.

**Evaluation Metrics**

Six common evaluation metrics (i.e., Precision (P), Recall I, F1-score, mean Average Precision (mAP), Model Parameter (Param.), and detection time) are used. These evaluation metrics utilized in our experiments include two parts: The localization accuracy and the complexity of the model. We use P, R, F1-score, and mAP to evaluate the classification and localization accuracy of the proposed method. We can calculate P, R, F1-score, and mAP in the image-patch level as Equations (17)–(20).

(17) $$P=\overset{C}{\underset{k=1}{\sum }}P_{k}$$

(18) $$R=\overset{C}{\underset{k=1}{\sum }}R_{k}$$

(19) $$mAP=\overset{C}{\underset{k=1}{\sum }}AP_{k}$$

(20) $$F1-score=\frac{2\times P\times R}{P+R}$$

in which,

(21) $$P_{k}=\frac{TP_{k}}{TP_{k}+FP_{k}}$$

(22) $$R_{k}=\frac{TP_{k}}{TP_{k}+FN_{k}}$$

(23) $$AP_{k}=\int_{0}^{1}P_{k}\left(R_{k}\right)\;dR_{k}$$

C is the number of defect classes on a fabric dataset. $TP_{k}$ refers to the number of real defect class k objects that are correctly detected as defective boxes. $FN_{k}$ refers to the number of real defect class k objects that are falsely detected as non-defective boxes (i.e., texture background) or other class boxes. And $FP_{k}$ refers to the number of real background area that is falsely detected as defective boxes.

The comprehensive metric mAP refers to the area enclosed by the P-R curve and the R axis. The F1-score metric is a comprehensive evaluator that uses both the R and P metrics. In addition, we use Param. Metric and detection time to quantitatively show the space and time complexity of the model.

### 4.2. Experimental Results and Discussion on TILDA Dataset

In order to test the ability of the method to detect defects on fabric images containing multiple texture backgrounds (including unpatterned background, regular patterned background, and irregular patterned background), we used the TILDA dataset for experiments.

#### 4.2.1. Experimental Settings

On the TILDA dataset, we divide the data set according to the ratio of 7:3, i.e., use 1117 images as the training set and 480 images as the testing set. The training steps of our method is 120 k; the batch size is 32; the optimizer is stochastic gradient descent optimizer (SGD); the momentum and weight decay are respectively set as 0.9 and 0.0005. The $\eta_{max}$, $\eta_{min}$, $T_{max}$ of cosine annealing scheduler are set as 1e-3, 0, 120 k, respectively. Considering the base models of the existing object detection-based fabric defect detection methods, we use a variety of common object detection models, including Faster RCNN (2015, NIPS), SSD (2016, ECCV), YOLOv3 (2018, CVPR), and FCOS (2019, CVPR) as comparison methods to show the advantages of the proposed method.

The configurations in our experiment are as follows:

- **Faster RCNN:** ResNet-50 backbone + FPN + RPN + SGD optimizer + StepLR scheduler +NMS.
- **SSD:** VGG-16 backbone + six layer feature pyramid + SGD optimizer + MultiStepLR scheduler + NMS.
- **YOLOv3:** DarkNet-53 backbone + three feature pyramid and feature fusion + Adam optimizer + NMS.
- **FCOS (anchor free detector):** ResNet-50 backbone + five layer FPN + NMS.
- **Original RefineDet:** VGG-16 backbone + four layer feature pyramid and head structure (ARM, TCB and ODM) +SGD optimizer + MultiStepLR scheduler + NMS.
- **Ours:** VGG-16 backbone + improved head structure (FCCA-ARM, BA-TCB and ODM) +SGD optimizer + Cosine annealing scheduler + DIoU-NMS.

#### 4.2.2. Results and Discussion

**Figure 10:**
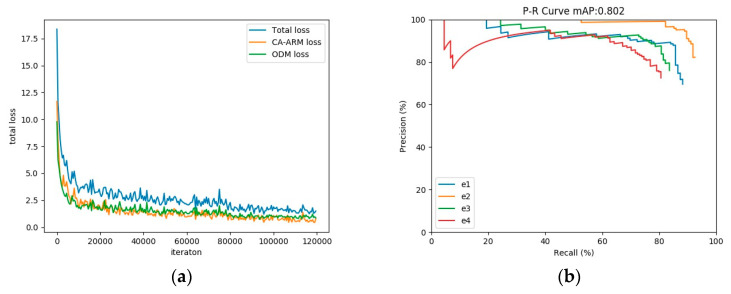
Experiment display of the proposed method on the TILDA dataset. (**a**) The loss of the proposed method on the TILDA dataset in the training stage; (**b**) The P-R curve of the proposed method on the TILDA testing set.

As shown in Figure 10a, on the TILDA dataset, the loss function of the proposed method continuously decreases during the training stage until convergence. After 120 k iterations, the training of model parameters is completed. Compared with five common detection methods, the proposed method has the highest defect localization accuracy, and the detection speed can reach real-time. As shown in Table 2 and Figure 10b, on comprehensive metrics F1-score, the proposed method is 21.7%, 17%, 35.4%, and 3.4% higher than Faster RCNN, SSD, YOLOv3, and FCOS, respectively. On the object detection benchmark metric mAP, the proposed method is 21.3%, 19.8%, 46.9%, and 3.4% higher than Faster RCNN, SSD, YOLOv3, and FCOS, respectively. In terms of detection speed, our method only adds a small amount of space and time cost compared with the original RefineDet. The parameter size of the entire model only reaches 43.1 million (i.e., only occupies about 168MB of disk space), and the detection time reaches 34 frames per second (FPS). These experimental results show that the proposed method achieves a trade-off between detection time and localization accuracy, and can efficiently and quickly detect various defects on the fabric image.

**Figure 11:**
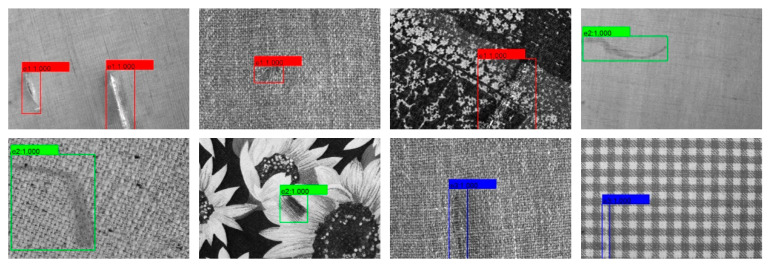

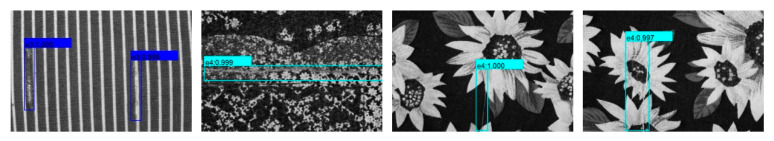
The detection result of the proposed method on the TILDA testing set.

As shown in Figure 11, the TILDA dataset contains eight different texture backgrounds, including unpatterned textures, regular patterned textures, and complex irregular patterned textures. For these challenging images of fabric defects, our method is effective mainly because of the good generalization ability of the object detection model. The object detection model mainly learns the feature of defect objects, rather than the background. Complex and diverse texture backgrounds may affect the detection accuracy of the model, but when there are enough training images with defect object, it also can be suitable for the fabric defect detection under different texture backgrounds. The results at the TILDA dataset demonstrate the advantages of our method on fabric images with complex texture backgrounds. Meanwhile, in Section 4.4, we will specifically show the results of the ablation experiment and the advantages of each component of the proposed method.

### 4.3. Experimental Results and Discussion on the Hong Kong Dataset and DAGM2007 Dataset

In order to demonstrate the good generalization ability of the proposed method, we use the Hong Kong Patterned Textures Database to verify the advantages of the proposed method on the small sample dataset and use the DAGM2007 dataset to show the robust defect detection ability of our method on the general texture defect dataset.

#### 4.3.1. Experimental Settings

In this experiment, we did not change the hyperparameters of the proposed method. The training steps is 120 k; the batch size is 32; the optimizer is SGD optimizer; the momentum and weight decay are respectively set as 0.9 and 0.0005. The $\eta_{max}$, $\eta_{min}$, $T_{max}$ of cosine annealing scheduler are set as 1e-3, 0, 120 k, respectively. On Hong Kong Patterned Textures Database, we divide the dataset according to the ratio of 6:4—i.e., 50 images are used as the training set, and 32 images are used as the testing set. Because there are a few defective images on the Hong Kong dataset, we mainly use the offline data augmentation methods to process the training data and expand the number of training set to 633. At the same time, interference operations (image brightness change, image blur, random flip, and random rotation) are added to images of the testing set to further verify the anti-interference ability of the model. On the DAGM2007 dataset, the number of training set images is 1046, and the number of images in the testing set is 1054. We mainly use online data augmentation methods to process training data.

#### 4.3.2. Results and Discussion

**Figure 12:**
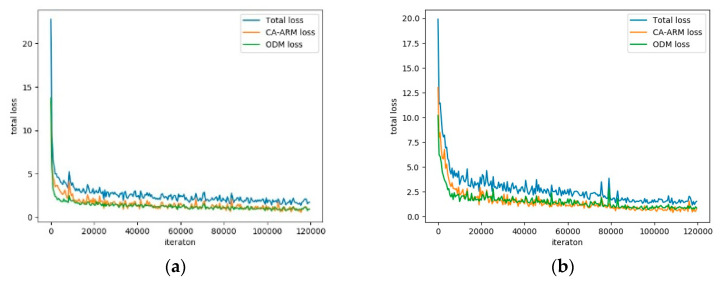
Loss function display of the proposed method on the Hong Kong dataset and DAGM2007 dataset. (**a**) The loss of the proposed method on the Hong Kong dataset in the training stage; (**b**) The loss of the proposed method on the DAGM2007 dataset in the training stage.

As shown in Figure 12, the loss function continuously decreases during the training stage until convergence. After 120 k iterations, the training of model parameters is completed.

**Figure 13:**
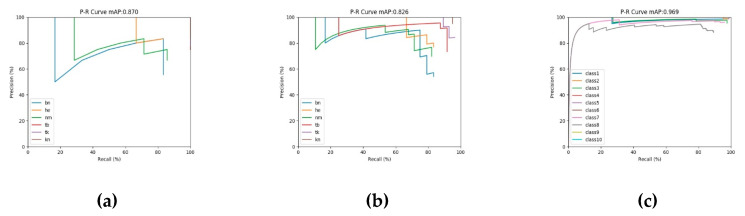
P-R curve display of the proposed method on the Hong Kong dataset and DAGM2007 dataset. (**a**) The P-R curve of the proposed method on Hong Kong testing set; (**b**) The P-R curve of the proposed method on Hong Kong testing set with interference; (**c**) The P-R curve of the proposed method on DAGM2007 testing set.

As shown in Table 3, the proposed method is 1.1% and 0.2% higher than baseline in mAP metric on Hong Kong testing set and DAGM2007 testing set, respectively, and in F1-score, the proposed method is 3.4% and 3.8% higher than the baseline, respectively, which shows our method can get a good trade-off between P and R metrics. We also applied interference operations, such as image brightness change, image blur, random flip, and random rotation, to the testing images of the Hong Kong dataset, increasing the number of testing images to 128. In the case of unchanged model parameters, the proposed method can still effectively detect defect areas on Hong Kong testing set with interference (82.6% mAP and 81.5% F1-score), which shows that the proposed method has a strong anti-interference ability. In particular, as shown in Figure 13, the P-R curve of the proposed method is close to the upper right corner, and the detection results (mAP value) for three testing sets are all higher than 82%. Meanwhile, our method consumes considerable time and space costs. When the model input is 320 ∗ 320, the detection time reaches high detection speed (higher than 18 FPS), and the model parameters only reach about 43 million in space, and only occupy about 168 MB of disk space.

**Figure 14:**
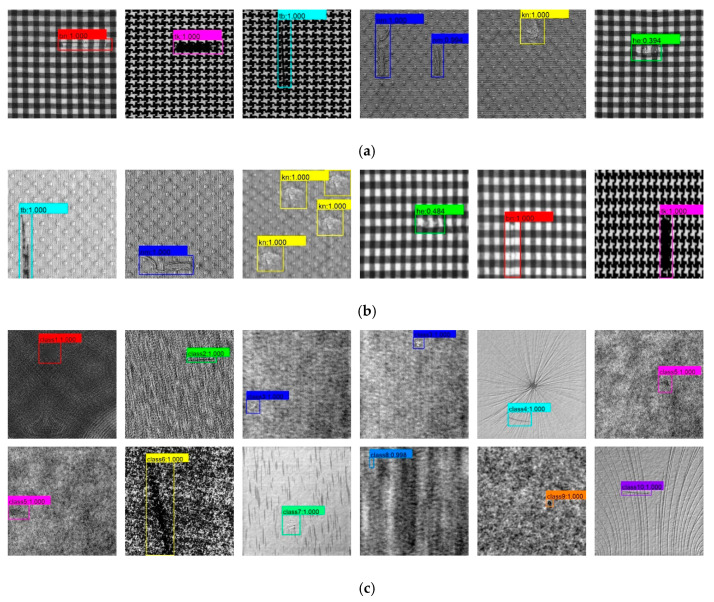
The partial detection result of the proposed method. (**a**) The detection result of the proposed method on Hong Kong testing set; (**b**) The detection result of the proposed method on Hong Kong testing set with interference; (**c**) The detection result of the proposed method on DAGM2007 testing set.

The experimental results on the Hong Kong testing set show that the proposed method obtains better detection results on the Hong Kong dataset. For this small sample data set, the defective samples of the training set are expanded by means of offline data augmentation. Under the premise that the generated images are close to the real images, the method in this paper is also applicable. Meanwhile, as seen in Figure 14a,b. The Hong Kong dataset is mainly periodic texture images of star type, box type, and dot type, which also verifies that our method is suitable for defect detection on periodic texture fabrics, even if there are many types of defects and a small number of samples in each defect class. As shown in Figure 14c, the DAGM2007 dataset mainly contains unpatterned background and simple irregular patterned background images. In this dataset, the proposed method achieves more than 96% in both mAP and F1-score metric. The experimental results on this dataset show that the proposed method is also suitable for general texture defect detection.

### 4.4. Ablation Experiments

In this part, on the one hand, we show the advantages of each component of the proposed method. On the other hand, we verify the impact of some general state-of-the-art optimization methods on the defect detection results.

**Figure 15:**
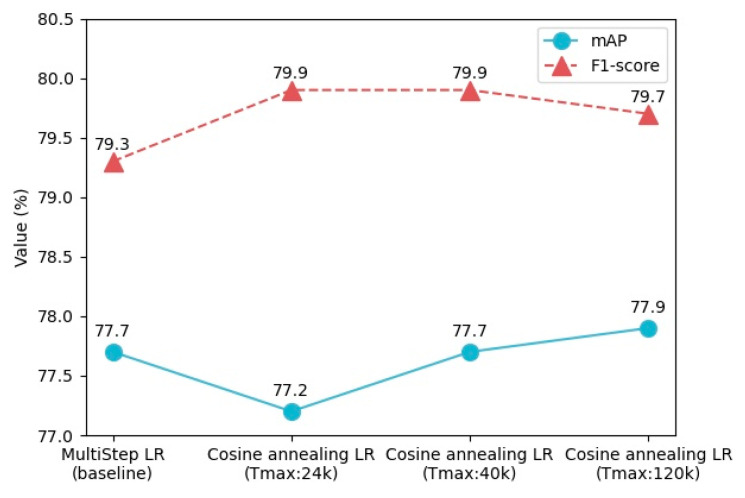
Detection results with different Tmax of Cosine annealing scheduler.

As shown in Table 4, it can be seen from experiments 1, 2, and 3 that the FCCA-ARM and BA-TCB structure in our model can effectively improve the localization accuracy of the model. The improved head structure makes the mAP metric increase by 2.2%, and the F1-score increases by 0.9%. Although the parameter amount of the model has increased slightly, it can also reach real-time detection. In addition, experiments 1, 4, and 5 prove that DIoU-NMS and cosine annealing optimization methods can boost the performance of the model without introducing extra computational cost. As shown in Figure 15, when the Tmax of the cosine annealing scheduler is set to 120 k (the maximum number of iterations), the mAP and F1-score of the model are better than the original RefineDet (baseline). The combination of these components and the optimization methods makes our model have higher detection accuracy and achieve a trade-off between localization accuracy and speed.

As shown in Table 5, we also verified the impact of other state-of-the-art general optimization methods, such as Swish activation function, Mish activation function, SE attention mechanism, SAM attention mechanism, GIoU regression loss, and DIoU regression loss on model performance. It can be seen from experiments 1, 2, and 3 that when replacing all the Relu activation functions of the backbone network, the Mish and Swish activation function does not greatly improve the model in the mAP metric. Experiments 1, 4, 5 prove that CAM works better in our model than SE and SAM. We also used GIoU loss and DIoU loss as odm_l loss; however, they made the localization accuracy decrease sharply, we still use Smooth L1 function as regression loss.

### 4.5. The Shortcomings and Outlook of The Proposed Method

**Figure 16:**
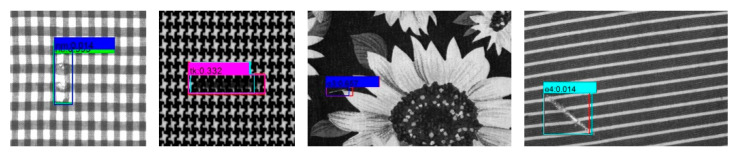
Some examples of false detected images in the experimental results.

The proposed method can detect the most defective areas efficiently and quickly. However, as shown in Figure 16, it is inevitable that some false predicted boxes and missed defect areas will be generated, mainly due to the highly similar shapes of some defect classes, the similar color of the defect, and the texture background.

In order to solve this problem, on the one hand, the defect classification capability of the model should be further improved in future work. On the other hand, the phenomenon of blur and uneven illumination during the image acquisition process should be avoided as much as possible. In order to reduce the cost of manual labeling and better meet the needs of the textile industry, we also consider introducing weakly-supervised object detection components in future work. Meanwhile, considering that Mixup, CutOut, and CutMix can effectively improve the classification ability of the model, we plan to use Mixup, CutOut, CutMix, and Mosaic augmentation methods to obtain more number of data samples for the training in future work.

## 5. Conclusions

A fabric defect detection method based on improved RefineDet is proposed in this paper. On the one hand, the proposed method uses RefineDet as the base model, and have an improved head based on the FCCA block and BA-TCB structure, which efficiently improve the defect localization accuracy of the model. On the other hand, the proposed method applies many general optimization methods, such as attention mechanism, DIoU-NMS, and cosine annealing scheduler, and verifies the effectiveness of these optimization methods in the fabric defect detection. Experimental results show that the proposed method is suitable for the defect detection of fabric images with unpattern background, regular patterns, and irregular patterns and can detect different fabric defects at high speed.

## Figures and Tables

**Table 1:** The configuration of the VGG-16 based backbone network.

Name	Layer	Output	Name	Layer	Output
Input		320∗320∗3	Conv4_2	3∗3-s1-p1, 512	40∗40∗512
Conv1_1	3∗3-s1-p1, 64 ^1^	320∗320∗64	Conv4_3	3∗3-s1-p1, 512	40∗40∗512
Conv1_2	3∗3-s1-p1, 64	320∗320∗64	Maxpool4	2∗2-s2-p0	20∗20∗512
Maxpool1	2∗2-s2-p0	160∗160∗64	Conv5_1	3∗3-s1-p1,512	20∗20∗512
Conv2_1	3∗3-s1-p1, 128	160∗160∗128	Conv5_2	3∗3-s1-p1,512	20∗20∗512
Conv2_2	3∗3-s1-p1, 128	160∗160∗128	Conv5_3	3∗3-s1-p1,512	20∗20∗512
Maxpool2	2∗2-s2-p0	80∗80∗128	Maxpool5	2∗2-s2-p0	10∗10∗512
Conv3_1	3∗3-s1-p1, 256	80∗80∗256	Conv6	3∗3-s1-p1,1024	10∗10∗1024
Conv3_2	3∗3-s1-p1, 256	80∗80∗256	Conv7	3∗3-s1-p1,1024	10∗10∗1024
Conv3_3	3∗3-s1-p1, 256	80∗80∗256	Conv8_1	1∗1-s1-p1, 256	10∗10∗256
Maxpool3	2∗2-s2-p0	40∗40∗256	Conv8_2	3∗3-s2-p1, 512	5∗5∗512
Conv4_1	3∗3-s1-p1, 512	40∗40∗512			

^1^ 3∗3-s1-p1, 64 means that the convolution layer with 3∗3 kernel size, 1 stride, 1 padding, and 64 output channel.

**Table 2:** Performance comparison of different object detection approaches on TILDA dataset.

Method	P (%)	R (%)	mAP (%)	F1-Score (%)	Parm.	Detection Time (FPS)
Faster RCNN	65.9	55.8	58.9	60.4	41.1 M	11.1
SSD	67.5	63.0	60.4	65.1	24.2 M	33.3
YOLOv3	59.7	38.4	33.3	46.7	63.0 M	19.8
FCOS	76.1	81.5	76.8	78.7	32.0 M	8.57
Original RefineDet	74.3	85.0	77.7	79.3	34.0 M	41.4
Ours	78.9	85.5	80.2	82.1	43.1 M	34.0

**Table 3:** Performance results on Hong Kong dataset and DAGM2007 dataset.

Dataset	Method	P (%)	R (%)	mAP (%)	F1-Score (%)	Parm.	Detection Time (FPS)
Hong Kong testing set (32)	Original RefineDet (baseline)	71.2	87.3	85.9	78.4	34.1 M	21.9
	Ours	73.6	92.1	87.0	81.8	43.2 M	18.5
Hong Kong testing set with interference (128)	Original RefineDet (baseline)	71.0	83.4	76.7	78.1	34.1 M	42.8
	Ours	76.6	88.7	82.6	81.5	43.2 M	30.1
DAGM2007 testing set (1054)	Original RefineDet (baseline)	96.6	97.5	96.7	97.0	33.2 M	45.2
	Ours	97.6	97.9	96.9	97.8	43.3 M	33.0

**Table 4:** Performance of components and optimization methods in our method.

Index	Experimental Settings	mAP (%)	F1-Score (%)	Parm.	Detection Time (FPS)
1	RefineDet (baseline)	77.7	79.3	34.0 M	41.4
2	RefineDet + FCCA-ARM	78.3	80.1	34.3 M	36.8
3	RefineDet + FCCA-ARM + BA-TCB	79.9	80.2	43.1 M	33.9
4	RefineDet + DIoU-NMS	77.7	80.0	34.0 M	39.7
5	RefineDet + Cosine annealing scheduler	77.9	79.7	34.0 M	40.9
6	Ours (RefineDet + FCCA-ARM + BA-TCB + DIoU-NMS + Cosine annealing scheduler)	80.2	82.1	43.1 M	34.0

**Table 5:** Performance of different other optimization methods.

Index	Experimental Settings	mAP (%)	F1-Score (%)	Parm.	Detection Time (FPS)
1	RefineDet (baseline)	77.7	79.3	34.0 M	41.4
2	RefineDet + Mish activation function	76.7	81.7	34.0 M	38.8
3	RefineDet + Swish activation function	76.2	78.7	34.0 M	37.6
4	RefineDet + SAM-ARM	77.7	79.1	34.3 M	40.2
5	RefineDet + SE-ARM	78.3	79.6	34.3 M	40.5
6	Ours + DIoU loss	71.5	68.8	43.1 M	33.6
7	Ours + GIoU loss	70.9	70.8	43.1 M	33.2

**Figure A1:**
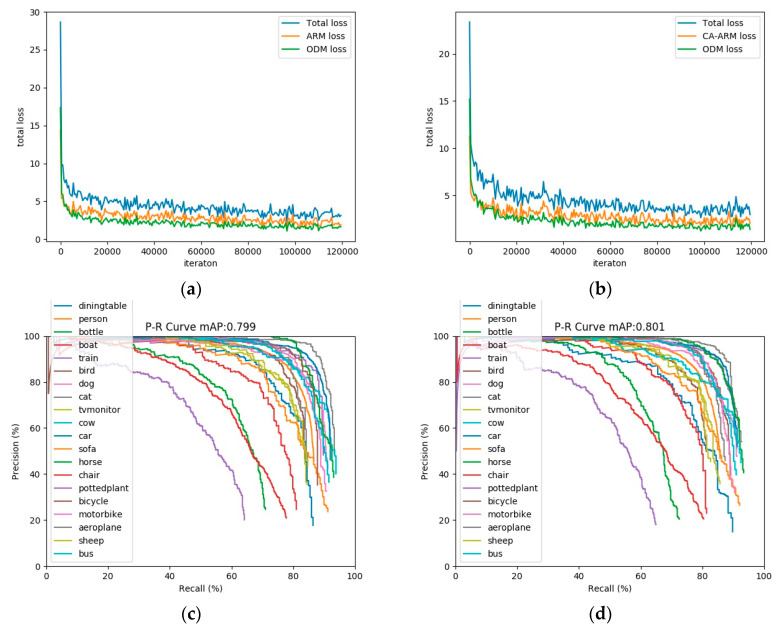
Loss function and P-R curve display on the VOC dataset. (**a**) The loss function of the original RefineDet; (**b**) The loss function of the proposed method; (**c**) The P-R curve of the original RefineDet; (**d**) The P-R curve of the proposed method.

## Appendix A

In order to further demonstrate the localization ability of the proposed method in the general object detection domain, we also use the PASCAL VOC dataset (https://pjreddie.com/projects/pascal-voc-dataset-mirror/)an object detection standard dataset, to show the performance of the proposed method.

### A.1. Experimental Settings

The network hyperparameters and data preprocessing methods are consistent with ones in Zhang et al. [34]—i.e., the backbone is VGG-16; the input size of the model is 320 × 320; the training steps is 120 k; the batch size is 32; the optimizer is SGD optimizer; the momentum and weight decay are, respectively, set as 0.9 and 0.0005. The PASCAL VOC dataset contains 20 common object classes that are diningtable, person, bottle, boat, train, bird, dog, cat, tvmonitor, cow, car, sofa, horse, chair, pottedplant, bicycle, motorbike, aeroplane, sheep, and bus. In this experiment, the training set is the VOC2007 trainval set (5011 images and 20 object classes) plus VOC2012 trainval set (11,540 images and 20 object classes). The testing set is the VOC2007 test set (4952 images and 20 object classes).

### A.2. Results and Discussion on PASCAL VOC Dataset

As shown in Figure A1b, after 120 k iterations, the training of our model parameters is completed. Meanwhile, as shown in Table A1, the mAP and F1-score metrics of the proposed method on the VOC2007 test set are 0.2% and 1.5% higher than the original RefineDet, respectively. Although the size and detection time of the proposed method increases a little, the localization accuracy of our model has been improved. On the premise of ensuring the real-time detection, our method achieves a trade-off between detection accuracy and detection speed. The results in this experiment show that our method can also be applied in the field of general object detection.

In addition, it can be seen from Figure A1c,d that compared with other classes, many object detectors have lower detection accuracy for bottle, chair, and pottedplant objects. For example, the APs for bottle, chair, and pottedplant classes are 63.0%, 62.6%, and 49.8%, respectively in our method; they are 61.4%, 62.2%, 49.3%, respectively in original RefineDet; they are 52.1%, 52.0%, 38.8% in original Faster RCNN (data comes from Table 6 in [13]); they are 47.6%, 54.7%, 48.6% in original SSD (data comes from Table 1 in [14], the input size of SSD is 300 × 300). This means that the proposed method is better than these common models in the detection of the bottle, chair, and pottedplant classes.

**Table A1:** Performance reults on PASCAL VOC2007 test set.

Method	P (%)	R (%)	mAP (%)	F1-Score (%)	Parm.	Detection Time (FPS)
Original RefineDet (Data come from Table 1 in [34])	-	-	80.0	-	-	40.3
Original RefineDet (Our reproduction in Pytorch)	35.9	86.0	79.9	50.7	34.4 M	37.2
Ours (RefineDet + FCCA-ARM + BA-TCB + DIoU-NMS + Cosine annealing scheduler)	37.4	86.3	80.1	52.2	43.6 M	29.2
